# Gibberellin Signaling through RGA Suppresses GCN5 Effects on Arabidopsis Developmental Stages

**DOI:** 10.3390/ijms25126757

**Published:** 2024-06-19

**Authors:** Christina Balouri, Stylianos Poulios, Dimitra Tsompani, Zoe Spyropoulou, Maria-Christina Ketikoglou, Athanasios Kaldis, John H. Doonan, Konstantinos E. Vlachonasios

**Affiliations:** 1 Department of Botany, School of Biology, Faculty of Sciences, Aristotle University of Thessaloniki, 54124 Thessaloniki, Greece; mpalouric@bio.auth.gr (C.B.); spoulios@bio.auth.gr (S.P.); dimitra.x.tsompani@gsk.com (D.T.); akaldis2003@hotmail.com (A.K.); 2 National Plant Phenomics Centre, Institute of Biological, Environmental, and Rural Sciences, Aberystwyth University, Gogerddan Campus, Aberystwyth SY23 3EE, UK; jhd2@aber.ac.uk; 3 Natural Products Research Centre of Excellence (NatPro-AUTh), Center of Interdisciplinary Research and Innovation of Aristotle University of Thessaloniki (CIRI-AUTh), 54124 Thessaloniki, Greece

**Keywords:** histone acetylation, plant development, stamen elongation, gibberellin, GCN5, ADA2b, RGA, DELLA, Arabidopsis, gibberellin biosynthesis

## Abstract

Histone acetyltransferases (HATs) modify the amino-terminal tails of the core histone proteins via acetylation, regulating chromatin structure and transcription. GENERAL CONTROL NON-DEREPRESSIBLE 5 (GCN5) is a HAT that specifically acetylates H3K14 residues. GCN5 has been associated with cell division and differentiation, meristem function, root, stem, foliar, and floral development, and plant environmental response. The flowers of *gcn5* plants display a reduced stamen length and exhibit male sterility relative to the wild-type plants. We show that these effects may arise from gibberellin (GA)-signaling defects. The signaling pathway of bioactive GAs depends on the proteolysis of their repressors, DELLA proteins. The repressor GA (RGA) DELLA protein represses plant growth, inflorescence, and flower and seed development. Our molecular data indicate that GCN5 is required for the activation and H3K14 acetylation of genes involved in the late stages of GA biosynthesis and catabolism. We studied the genetic interaction of the RGA and GCN5; the RGA can partially suppress GCN5 action during the whole plant life cycle. The reduced elongation of the stamen filament of *gcn5–6* mutants is reversed in the *rga–t2;gcn5–6* double mutants. RGAs suppress the GCN5 effect on the gene expression and histone acetylation of GA catabolism and GA signaling. Interestingly, the RGA and RGL2 do not suppress ADA2b function, suggesting that ADA2b acts downstream of GA signaling and is distinct from GCN5 activity. In conclusion, we propose that the action of GCN5 on stamen elongation is partially mediated by RGA and GA signaling.

## 1. Introduction

For the activation of gene expression during development, transcription factors must overcome a repressive chromatin structure, which is accomplished with the help of multiprotein complexes [1,2]. One class of complexes modify the nucleosomal histones through acetylation, phosphorylation, methylation, and other modifications [3]. The acetylation of specific lysine residues in histone N-terminal tails is catalyzed by histone acetyltransferases (HATs), which are involved in transcriptional regulation and other nuclear processes. HATs are part of large multiprotein complexes, like the SAGA complex, in which their activity is enhanced, their substrate specificity is modified, and the whole complex is recruited to target sequences on the genome with the help of other components involved in protein–protein interactions [4]. HATs and HDACs (histone deacetylases) can target promoters to activate or suppress gene expression, respectively.

GENERAL CONTROL NON-DEREPRESSIBLE 5 (GCN5, also known as HAG1) is a histone acetyltransferase [5,6] involved in many developmental processes and responses to environmental stimuli [4,7]. The gcn5 mutants exhibit pleiotropic phenotypes, including dwarfism, loss of apical dominance, upward curled and serrated leaves, abnormal inflorescence meristem, abnormal flower development, and shorter roots [8,9,10,11,12,13]. GCN5 is the catalytic subunit of the Arabidopsis SAGA (Spt–Ada–GCN5–acetyltransferase) complex [4,7,14,15] that acetylates H3 and H2A in nucleosomes and has been shown to interact with the transcriptional adaptors ADA2a and ADA2b [5,16,17]. The ada2b mutants present a phenotype highly similar but not identical to gcn5 mutants [9,18,19]. The *ada2b* mutants are characterized by dwarfism, small dark green curled leaves, and infertility [9]. GCN5 and ADA2b affect gynoecium development by modulating auxin and cytokinin signaling [20]. Furthermore, the loss of GCN5 function affects stamen elongation, especially in flowers formed early in development [10]. GCN5 and ADA2b integrate diverse internal and external signals into plant responses, including light, temperature, and biotic and abiotic stresses [4]. For instance, GCN5 targets the heat-stress response genes HSFA3 and UVH6 and induces their expression, increasing RNA polymerase II engagement and H3K9/14 acetylation levels, suggesting that GCN5 could be a target of several stress-inducible transcription factors or other chromatin-related factors [21].

Plant hormones, specifically gibberellins, modulate stamen development and function [22]. Gibberellin (GA) biosynthetic mutants revealed that GAs stimulate stamen filament elongation through increased cell elongation and promote anther dehiscence [23]. In Arabidopsis plants, GA is perceived by three GA receptors: gibberellin insensitive dwarf 1s (GID1a, GID1b, and GID1c) receptors. The triple GA insensitive mutant produces a more severe stamen phenotype than the mutant in GA biosynthesis, *ga1-3* [24]. The binding of GA to these receptors promotes the interaction with DELLA proteins, which are GRAS domain proteins and major repressors of GA signaling [25]. In Arabidopsis plants, five DELLA proteins have been identified: GA insensitive (GAI), repressor of GA1-3 (RGA) and RGA-like (RGL1, RGL2, and RGL3) proteins. The binding of DELLA proteins to the GA–GID1 complex results in polyubiquitination and triggers their degradation by the 26S proteasome [26]. Several transcription factors have been identified downstream of DELLA proteins, including members of PIFs and MYB families [27,28]. Specifically, MYB21 and MYB24 control stamen filament growth by acting downstream of DELLA proteins [29].

In this study, we explored the role of GCN5 in gibberellin responses and, using genetic and molecular approaches, demonstrated that the RGA partially suppress the GCN5 effect on stamen elongation by affecting the H3 acetylation on genes involved in gibberellin biosynthesis, catabolism, and signaling.

## 2. Results

### 2.1. ADA2a, ADA2b and GCN5 Are Required for the Hypocotyl Response to Exogenous GA

**Figure 1:**
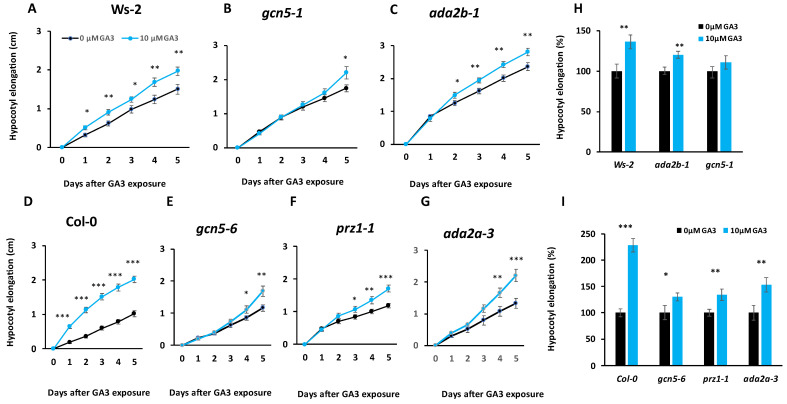
The effect of GA3 on hypocotyl elongation of Ws-2 (**A**), *gcn5-1* (**B**), *ada2b-1* (**C**), Col-0 (**D**), *gcn5-6* (**E**), *prz1-1 (***F**), and *ada2a-3* (**G**) seedlings. Sensitivity of Ws-2, *gcn5-1* and *ada2b-1* (**H**), and Col-0, *gcn5-6*, *prz1-1*, and *ada2a-3* (**I**) mutant seedlings after four days of exposure to 10 μΜ GA_3_. Asterisks *, **, and *** indicate statistically significant differences from 0 μΜ GA3 using Student’s *t*-test for *p* < 0.05, *p* < 0.01, and *p* < 0.001, respectively (*n* = 90 plants).

Hypocotyl elongation is affected in SAGA-related mutations [9] and is a GA-sensitive process. Therefore, we hypothesized that mutant hypocotyls might display altered responses to exogenous GA. The effect of gibberellins on the hypocotyl elongation of *ada2a*, *ada2b*, and *gcn5* mutant seedlings was measured for five consecutive days after applying 10 µM GA_3_. Initially, applying gibberellins increased the hypocotyl growth in both Ws-2 and Col-0 wild-type seedlings (Figure 1A,D). The response was more significant in Col-0 seedlings than in Ws-2 seedlings. The hypocotyl growth in *gcn5-1* and *gcn5-6* mutants was slower than in wild-type plants, and the response to gibberellins was minor and measurable after four days of exposure (Figure 1B,E). The sensitivity of both *gcn5* mutants to GA_3_ was reduced (Figure 1H,I), suggesting that GCN5 is required for GA-induced hypocotyl elongation. The response of two *ada2b* mutant alleles, *ada2b-1* and *prz1-1*, to gibberellins was also lower than that of the wild-type plants but with a shorter delay of two days (Figure 1C,F). The sensitivity to GA_3_ of both *ada2b* mutants was also reduced (Figure 1H,I), implying that ADA2b is also essential for GA-induced hypocotyl growth. In contrast, *ada2a-3* mutants displayed an increased hypocotyl elongation upon GA treatment, albeit lower than wild-type plants (Figure 1G). The sensitivity of *ada2a-3* was higher than *gcn5* and *ada2b* mutants but lower than the wild-type plants (Figure 1I), suggesting that ADA2a has a minor role in GA-induced hypocotyl growth.

### 2.2. The Role of ADA2a, ADA2b, and GCN5 in the Root Elongation of Seedlings after Exposure to Gibberellins

Root growth is affected by GCN5 and ADA2b [9,30]. Root elongation depends on the action of gibberellins [31]. The effect of gibberellins on the primary root elongation of *ada2a*, *ada2b*, and *gcn5* mutant seedlings was measured for 4 or 5 consecutive days after applying 10 µM GA3. The exposure to gibberellins slightly decreased the root growth in both Ws-2 and Col-0 wild-type seedlings (Figure S1). The response of *ada2b-1* and *prz1-1* roots to gibberellins was similar to that of the wild-type seedlings (Figure S1). In contrast, the roots of *gcn5-1* mutants were more responsive to gibberellin than the wild-type seedlings (Figure S1). Finally, the root growth of *ada2a-3* seedlings decreased upon exposure to GA_3_, which was similar to *gcn5* mutants, indicating that ADA2a is also implicated as a negative regulator of GA-induced root growth retardation (Figure S1).

### 2.3. ADA2b and GCN5 Affect Flower Morphology by Modulating Gibberellin Biosynthesis

**Figure 2:**
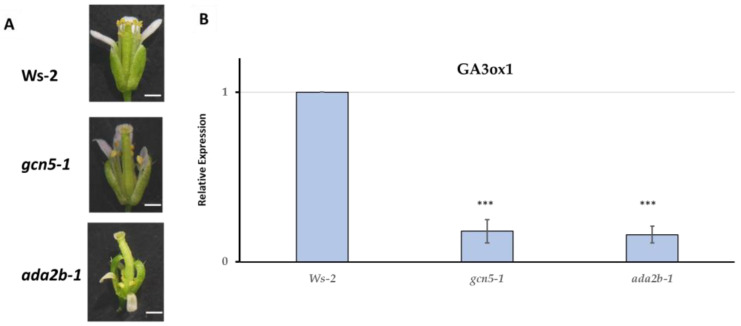
GCN5 and ADA2b affect flower development. (**A**) Early formed flower in *gcn5-1*, *ada2b-1*, and wild-type Ws-2; bar equals 0.5 cm. (**Β**) *GA3ox1* expression in flowers of *gcn5-1*, *ada2b-1*, and wild-type. Asterisks *** indicate statistically significant differences from Ws-2 for *p* < 0.001, respectively.

Arabidopsis mutants in GCN5 and ADA2b are characterized by abnormal flower development [9]. Both mutant plants displayed short stamens, especially in the early formed flowers (Figure 2A). The stamen elongation is restored only in the late-forming gcn5 flowers [9,10]. Defects on gibberellin biosynthesis or signaling could raise this effect [24]. Therefore, we monitored the expression of GA3ox1, the last enzyme in GA biosynthesis, in the *ada2b-1* and *gcn5-1* early formed flowers. Indeed, the expression of GA3ox1 was dramatically reduced in both mutants (Figure 2B), suggesting that GCN5 and ADA2b act as positive regulators of GA biosynthesis gene expression in early formed flowers in Arabidopsis plants. As a result, we hypothesized that GCN5 and ADA2b regulate GA biosynthesis in flowers through GA signaling components, including DELLA proteins. The RGA, RGL1, and RGL2 DELLA proteins are known to be required for flower development [32] and to be involved in the regulation of GA biosynthesis [33]. Therefore, a genetic approach was carried out by crossing *gcn5-6 (hag1-6)* and *ada2b-1* null mutants with *rga-t2* mutants to test the proposed regulation in flowers and hypocotyl elongation.

### 2.4. Mutation in RGA Partially Suppresses gcn5 Phenotypes

#### 2.4.1. Hypocotyl Elongation Is Restored in rga;gcn5 Double Mutants

**Figure 3:**
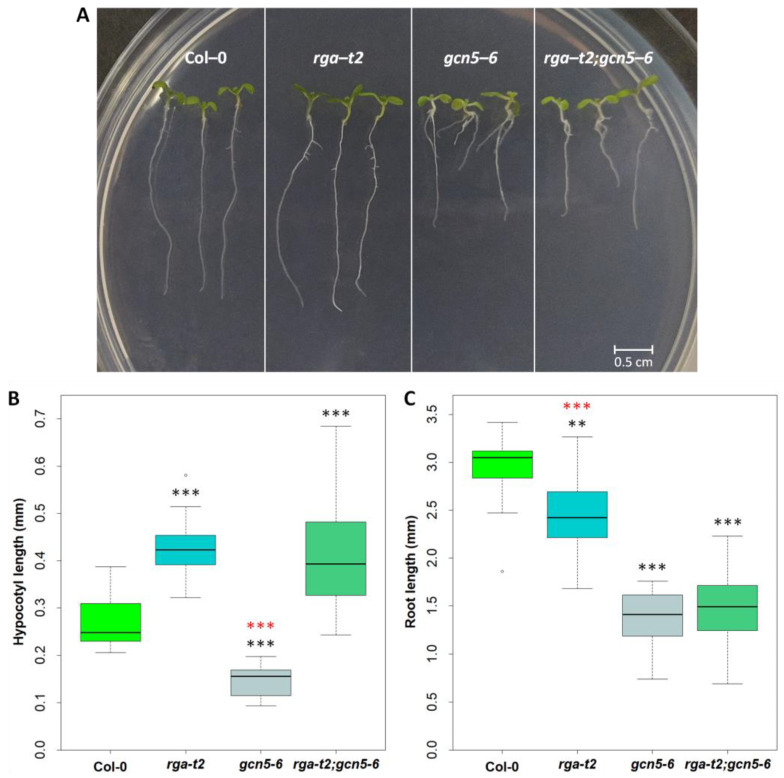
Characterization of *rga-t2;gcn5-6* double mutants at seedlings stage. (**A**) Seedlings of Col–0, *rga–t2*, *gcn5–6*, and *rga–t2; gcn5–6*, at day 7 of growth. The length of the hypocotyl (**B**) and root (**C**) of seven-day-old seedlings. Asterisks ** and *** indicate statistically significant differences from Col–0 for *p* < 0.01 and *p* < 0.001, respectively. Similarly, divergence from the *rga–t2;gcn5–6* double mutant is shown with red asterisks. Error bars represent the standard deviation.

In young seedlings, the gcn5–6 mutant showed reduced hypocotyl elongation (Figure 3A,B), whereas an elongated hypocotyl was observed in the *rga-t2* seedlings, compared to the wild-type seedlings (Col-0). In contrast, in the *rga–t2;gcn5–6* double mutant, the hypocotyl phenotype resembled *rga–t2*, completely reversing the hypocotyl length of the *gcn5–6* mutant, suggesting that the RGA could suppress the effect of GCN5 on hypocotyl elongation. The root growth in the *gcn5–6* mutant was significantly reduced compared to the wild-type and *rga-t2* mutant plants (Figure 3A,C). In the *rga–t2;gcn5–6* mutant, the root length was similar to gcn5 root growth. Therefore, the RGA represses the GCN5 action on hypocotyl elongation in the light, acting downstream of GCN5. In contrast, GCN5 promotes root elongation independently from the RGA.

#### 2.4.2. GCN5 Is Required for the Inflorescence Growth and Is Partially Suppressed by Loss of RGA

**Figure 4:**
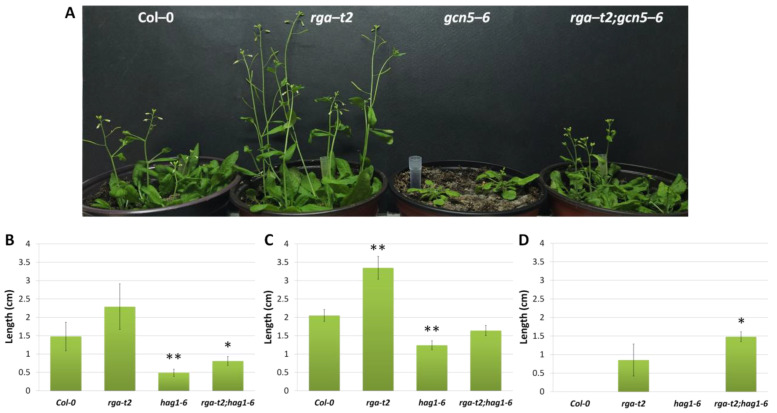
Col-0, *rga-t2*, *gcn5-6*, and *rga-t2;gcn5-6* mutant plants after 36 days of growth (**A**). The effect of RGA and GCN5 on first internode length (**B**), second internode length (**C**), and third internode length (**D**). Asterisks * and ** in B and C indicate statistically significant differences from Col–0 for *p* < 0.05 and *p* < 0.01, respectively, and in D, the asterisk indicates a significant difference from *rga-t2* for *p* < 0.05. Error bars represent the standard deviation (*n* = 16).

During the vegetative stage, the *gcn5–6* mutant exhibits a distinct phenotype with small serrated leaves. Interestingly, even after 20 days of growth, the rosette leaves of the *rga–t2;gcn5–6* double mutant plants remained serrated, indicating that the RGA does not play a significant role in the GCN5 effect on leaf development (Figure S2). In later development, *gcn5–6* mutant plants are characterized by delayed flowering as previously described [34] and short inflorescence relative to wild-type Col–0 plants. In contrast, *rga–t2* plants flower earlier and show longer inflorescence than wild-type plants. In the *rga–t2;gcn5–6* double mutant plants, the inflorescence growth is partially restored, compared to *gcn5–6*, without reaching the growth rate of Col–0 (Figure 4A). After the opening of the first flower, at 30–35 days of age for the wild-type, *rga*, and *rga–t2;gcn5–6* mutants and ∼50 days for the *gcn5–6* mutant, the number of lateral inflorescences and the length of internodes were measured. Two lateral inflorescences are identified in wild-type plants and the *gcn5–6* mutant. The *rga–t2* and *rga–t2;gcn5–6* mutants have two or three lateral inflorescences, suggesting that the RGA regulates the number of lateral inflorescences in Arabidopsis plants (Table S1). As shown in Figure 4B, the length of the first internode, which refers to the basal part of the inflorescence starting from the rosette to the first axillary bud, is shorter in the *gcn5-6* mutant than the Col-0 and *rga–t2* mutant plants. In the *rga–t2;gcn5–6* double mutant, the length of the first internode is noticeably shorter than Col–0 and *rga–t2*, while it does not show a statistically significant difference from the *gcn5–6* mutant. Therefore, *rga-t2* can not suppress the *gcn5* defect for the first internode. The second internode, which concerns the part of the inflorescence between the first and second cauline leaf, is longer in the *rga–t2* compared to Col–0, while *gcn5–6* again shows a reduction in length. In the double mutant, however, the length of the second internode is greater than in *gcn5–6* mutant plants (Figure 4C). These results suggest that GCN5 is required to properly elongate the internodes in the inflorescence growth and is partially suppressed by the RGA. A third internode is found only in the *rga–t2* and *rga–t2;gcn5–6* mutants. The length of the third internode appears to be slightly elongated in the double mutant (Figure 4D).

After two months, Col–0, *rga–t2*, and double mutant plants completed their life cycle while the *gcn5–6* mutant continued growing. The final height of *rga–t2* plants is significantly higher than Col–0 plants. In contrast, the *gcn5–6* mutant plants are still flowering, extending their lifespan. The *rga–t2;gcn5–6* double mutant shows a slightly longer life cycle than wild-type plants but shorter than *gcn5–6* plants. Its final main inflorescence length reaches that of the wild-type plants, completely reversing the developmental problem observed in the gcn5–6 mutant plants (Figures S3 and S4A), suggesting that the RGA is required for the GCN5 action in the latest stages of plant development. At the third month of growth, *gcn5–6* plants do not grow in height; however, they become very bushy (Figure S3) due to numerous secondary inflorescences (Table S2). This phenotype is reversed in the double mutant, indicating that the RGA is required for the GCN5 regulation on secondary inflorescence formation.

#### 2.4.3. RGA Is Required for GCN5 Function on Stamen Elongation in Early Flowers

**Figure 5:**
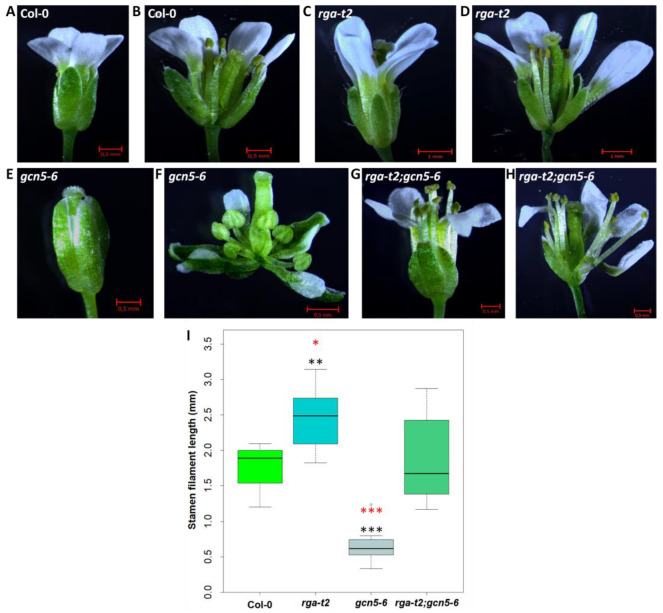
The effect of GCN5 and RGA on first-formed flowers. Closed and open flower Col–0 (**A**,**B**), *rga–t2* (**C**,**D**), *gcn5–6* (**E**,**F**), and *rga–t2;gcn5–6* (**G**,**H**). Stamen filament length (**I**). Asterisks *, **, and *** indicate the statistically significant difference in filament length from Col–0 for *p* < 0.05, *p* < 0.01, and *p* < 0.001, respectively. Similarly, red asterisks show statistically significant differences from the stamen growth of the double mutant. Error bars represent the standard deviation.

During inflorescence development, the early flowers of the *gcn5–6* mutants have short petals that do not exceed the length of the sepals (Figure 5E,F). The number of stamens remains the same as in the wild-type mutants (Table S3). However, the length of the stamen filament is significantly shorter (Figure 5I), which is associated with reduced fertility, suggesting that GCN5 is a positive regulator of stamen filament growth. In the *rga-t2* mutant, the number of stamens vary between five and six (Table S3) and the length of the stamen filament is increased compared to wild-type flowers (Figure 5A–D,I). In the double mutant, the petal length phenotype of *gcn5–6* is wholly reversed (Figure 5G,H). The number of stamens (Table S3) and filament length (Figure 5I) displayed significant variation, but most flowers had more stamens than Col–0 and the filament length was fully restored compared to wild-type flowers. These results indicate that removing the RGA fully compensates for the loss of GCN5 on stamen elongation.

The *gcn5–6* plants showed male sterility, which prevents the flower from self-fertilising. Effectively, no siliques and seeds are produced. In contrast, silique development and seed production are achieved in the *rga–t2;gcn5–6* double mutant. This result suggests that the removal of the RGA can compensate for the loss of GCN5, leading to successful silique growth and seed production. However, the double mutant siliques are significantly shorter than those of the wild-type and *rga* mutants, suggesting a positive role of GCN5 on silique growth or pollen fertility (Figure S5).

### 2.5. The Effect of GCN5 and RGA on Gene Expression in Hypocotyls and Flowers

Then, to further explore the effect of GCN5 and the RGA in hypocotyl growth, we monitored the expression of genes involved in the biosynthesis, catabolism, and signaling of gibberellins in the hypocotyls and cotyledons. *GA3ox1*, the last gene in the GA biosynthesis pathway, which is highly expressed in cotyledons and the apical meristem [35], showed an increased expression in *gcn5–6*, while in the *rga–t2;gcn5-6* double mutant, the expression was partially reversed (Figure S6a). The GA2ox family of proteins participates in the catabolism of gibberellins. It is known that the *GA2ox4* gene is expressed in the shoot apical meristem, the *GA2ox6* gene is expressed in the stem and vessels, and the *GA2ox8* gene is expressed in the leaf stomatal cells [36]. *GA2ox4* expression was increased in all mutants tested, with *gcn5–6* showing a four-fold increase from the wild type. In *rga–t2;gcn5–6*, the *GA2ox4* expression was reversed close to the levels of the *rga–t2* mutant (Figure S6b). The expression of the *GA2ox6* gene did not change significantly in the *rga–t2* and *gcn5–6* mutants from the wild type, while in the double mutant, an increase was observed, suggesting a synergistic action of the RGA and GCN5 (Figure S6c). Finally, the *GA2ox8* gene displayed an increased expression in *gcn5–6* and the double mutant *rga–t2;gcn5–6* (Figure S6d). The GA receptor, GID1b, showed a slightly increased expression in the single mutants and a three-fold upregulation in *rga–t2;gcn5–6* (Figure S6f), while no significant difference was observed in the expression of GID1a between the genotypes (Figure S6e). GAI expression in rosette leaves was slightly decreased in *gcn5-1* and *ada2b-1* mutants, while there was no detectable change in RGA expression [9,37]. In hypocotyls, the expression of GAI was slightly increased in the single and double mutants compared to wild-type mutants (Figure S6g). RGL2 showed a reduced expression in the *gcn5–6* mutant, which was reversed in the double mutant and upregulated compared to the wild-type and the *rga–t2* mutant (Figure S6h).

**Figure 6:**
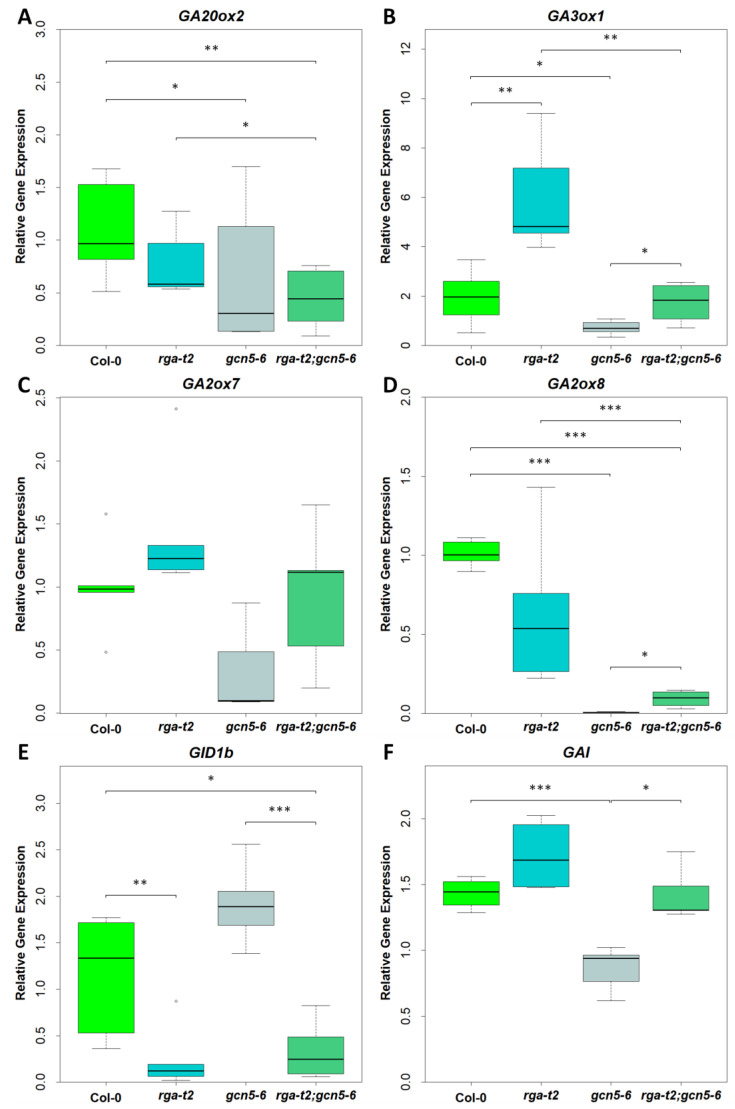
Expression of gibberellin biosynthesis genes *GA20ox2* (**A**) and *GA3ox1* (**B**), gibberellin catabolism genes *GA2ox7* (**C**) and *GA2ox8* (**D**), GA-receptor *GID1b* (**E**), and *GAI* DELLA gene (**F**) in early flowers of *Arabidopsis thaliana* inflorescence. Error bars show the standard deviation. Asterisks show statistical significance compared to Col–0 and *rga–t2;gcn5–6* double mutant, using Student’s *t*-test: *, *p* < 0.05, **, *p* < 0.01, and ***, *p* < 0.001.

Using real-time RT-PCR, we studied the gene expression in gibberellin biosynthesis, catabolism, and signaling in the early flowers. As shown in Figure 6A, *GA20ox2* is expressed at a lower level in the flowers of gcn5–6 mutants in comparison with Col–0 and *rga–t2*, while in *rga–t2;gcn5–6*, a partial restoration of expression levels is found, but it is not significant. The *GA3ox1* gene is expressed in the base of the young flower, apex, and sepal vessels and mainly in the filament of the stamens of the mature flower [35,38]. As shown in Figure 6B, a decreased expression in *gcn5–6* flowers was observed, whereas it has increased expression in *rga–t2* flowers compared to the wild-type flowers. In the double mutant, the expression of *GA3ox1* was higher than *gcn5-6*, close to wild-type levels. Therefore, the RGA appears to reverse the expression levels of *GA3ox1* in the *gcn5* background. Regarding the genes expressing GA catabolism enzymes, *GA2ox7* and *GA2ox8* are expressed in the whole flower [36]. The *GA2ox7* gene is not expressed in *gcn5–6* early formed flowers (Figure 6C). In the double mutant, *GA2ox7* expression is detectable at a lower level than wild-type or *rga* mutants. Similarly, *GA2ox8* expression is deficient in *gcn5–6* flowers compared to wild-type or *rga* mutants, while it is increased slightly in the *rga–t2;gcn5–6* double mutant (Figure 6D). The expression of the GA receptor, GID1b, increased in the *gcn5-6* mutants compared to wild-type plants. In the *rga* mutant early flowers, GID1b was decreased to low levels. A decreased expression of *GID1b* in the double mutant was also observed (Figure 6E). Therefore, the RGA reverses the effect of GCN5 on GID1b expression levels. The reduced gene expression of the GAI DELLA protein is observed in the early formed flowers of the *gcn5–6* mutant. The GAI expression in the flowers of *rga–t2;gcn5–6* mutants does not show a statistically significant deviation from the wild-type mutants (Figure 6F). Therefore, the positive regulation of GAI expression by GCN5 in primary flowers appears to be reversed by the action of the RGA DELLA protein.

### 2.6. GCN5 and RGA Alter H3K14 Acetylation in the Promoter of GA-Related Genes

**Figure 7:**
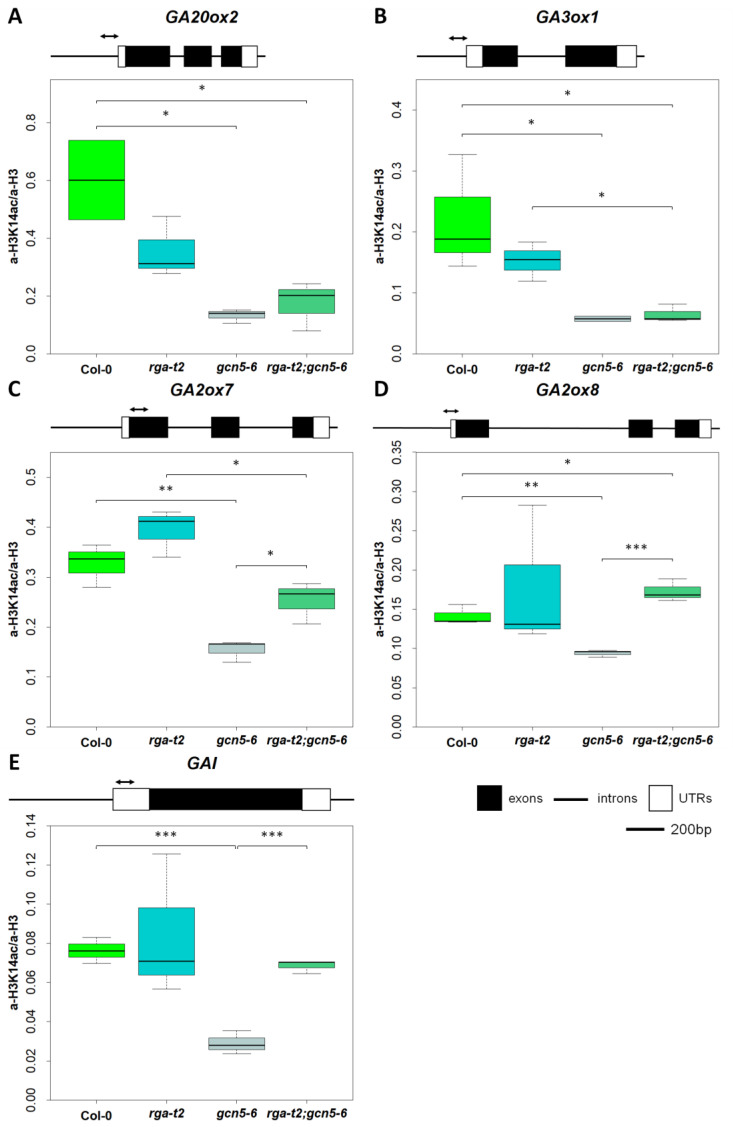
The acetylation status of histone 3 lysine 14 (H3K14) in the gibberellin biosyntheses genes *GA20ox2* (**A**) and *GA3ox1* (**B**), gibberellin catabolism genes *GA2ox7* (**C**) and *GA2ox8* (**D**), and DELLA gene *GAI* (**E**). Col–0, *rga–t2*, *gcn5–6*, and *rga–t2;gcn5–6* inflorescences were harvested for chromatin immunoprecipitation using antibodies against acetylated H3K14 and H3. Error bars show the standard deviation. Asterisks show statistical significance compared to Col–0 and *rga–t2;gcn5–6* double mutant, using Student’s *t*-test: *, *p* < 0.05, **, *p* < 0.01, and ***, *p* < 0.001.

To examine whether the observed changes in gibberellin-related gene expression in the *gcn5-6* and the *rga-t2;gcn5-6* double mutant resulted from changes in the acetylation status of its locus, we performed a ChIP analysis using antibodies for total histone H3 and acetylated lysine 14 in histone H3 (H3K14); H3K14 is known as the GCN5 target for acetylation [39,40]. Our results showed that in the promoter region GA20ox2 and GA3ox1 loci, total histone H3 acetylation is reduced significantly in gcn5-6 compared to wild-type plants (Figure 7A,B). In the double mutant, H3K14 acetylation levels were also low, suggesting that H3K14 acetylation levels correlate with the gene expression profile. Thus, the results suggest that GCN5 is required for the H3K14 acetylation of late GA biosynthetic genes, consistent with the altered expression levels. Then, we explored the effect of GCN5 and the RGA on the H3 acetylation in two genes involved in GA-catabolism, GA2ox7 and GA2ox8. The H3K14 acetylation level was almost undetectable in the *gcn5-6* plants compared to wild-type and *rga-t2* plants (Figure 7C,D). In the double mutants, the level of H3K14 acetylation was restored to wild-type levels. These results suggest that H3K14 acetylation in the GA-catabolism genes is affected through the RGA pathway. A similar scenario is observed in the 5-UTR region of the GAI DELLA protein; the level of H3K14 acetylation is low in the *gcn5-6* inflorescences compared to wild-type and *rga-t2* inflorescences (Figure 7E). H3K14 acetylation returns to wild-type levels in the double mutant, again correlated with the gene expression profile. This result suggests that the loss of RGA action restores the GCN5 requirement for both the expression and histone acetylation of GAI.

### 2.7. Mutation in RGA Did Not Suppress ada2b Phenotypes

In Arabidopsis plants, non-functional *ada2b* mutants exhibit developmental problems such as dwarfism, delayed root growth, flowers with short petals and stamens, increased infertility [9], and the reduced expression of GA biosynthesis genes (Figure 2B). Many of these phenotypes are similar to *gcn5* mutants, since ADA2b is physically associated with GCN5 [16] and could be related to plant responses to GA, suggesting potential problems in GA signaling. Therefore, we characterize *ada2b-1;rga-t2* and *ada2b-1;rgl2-1* double mutants and *ada2b-1;rga-t2;rgl2-1* triple mutants to explore if the RGA alone or with RGL2 could partially suppress the *ada2b* phenotype. The Supplemental Figure S7a clearly shows that the triple mutant *ada2b-1;rga-t2;rgl2-1* and the double mutants *ada2b-1;rga-t2* and *adab-1;rgl2-1* display a phenotype similar to that of *ada2b-1*, characterized by a short root length compared to the wild-type seedlings. In addition, the mutants have an increased number of secondary roots and elongated hypocotyls. Later in the plant development, during the flowering period, both the double mutants *ada2b-1;rga-t2* and *ada2b-1;rgl2-1* and the triple mutant *ada2b-1;rga-t2;rgl2-1* display a dwarf phenotype similar to *ada2b-1* plants (Supplemental Figure S7b). The inflorescence elongation was severely limited relative to the wild-type plants. These results indicate that the absence of the RGA and RGL2 does not reverse the *ada2b-1* phenotype. Therefore, ADA2b function is not suppressed by the RGA, suggesting a distinct regulation of ADA2b and GCN5 in the GA signaling pathway.

## 3. Discussion

This manuscript explored the role of the histone acetyltransferase GCN5 and the transcriptional adaptor ADA2b in gibberellin responses. We found that GCN5 regulates the expression of genes involved in GA biosynthesis, catabolism, and signaling in Arabidopsis plants by modulating the histone acetylation. Furthermore, we showed that the RGA DELLA protein partially suppresses the GCN5 function in flower development and stamen elongation independent from ADA2b.

Histone acetyltransferase GCN5 and the associated coactivator ADA2b are involved in diverse developmental processes, including root growth, cell elongation, trichome differentiation, floral initiation, apical meristem function, and floral reproduction [7]. Many of those processes could arise from defects in GA signaling. Gibberellins affect many biological processes, including seed germination, cell elongation, transition to flowering, and flower development [41]. Our data suggest that GCN5 affects histone acetylation (H3K14Ac) in the promoter of genes involved in the last step of GA biosynthesis and members of GA2 oxidases involved in GA catabolism. These effects are correlated with the gene expression, suggesting that GCN5 is a positive regulator of GA homeostasis in the early formed flowers. The effect of GCN5 on GA-inactivating genes is tissue and gene-specific since GA2ox4 and GA2ox8 are upregulated in *gcn5* mutants, whereas GA2ox6 expression was downregulated in hypocotyls. GA2ox6 was suggested as a target of the PAGA complex, which contains GCN5 and ADA2a in 12-day-old seedlings [42].

Without gibberellins, the DELLA proteins positively regulate the late GA biosynthesis genes and the receptor family GID1, whereas they negatively regulate the GA-inactivating genes [32]. Therefore, we genetically explored the effect of the RGA on GCN5 function by characterizing the double mutants. Our data suggest that the RGA could partially suppress many gcn5 phenotypes during the life cycle of Arabidopsis plants. In the seedlings, the RGA suppresses the positive role of GCN5 on hypocotyl elongation, which is correlated with the effect on GA3ox1, GA2ox4, and RGL2 gene expression. During reproductive stages, the RGA suppresses the GCN5 effect on the primary inflorescence growth, especially in the late developmental stages and the formation of secondary inflorescences, restoring the bushy appearance of *gcn5* mutants and suggesting that the RGA mediates the positive role of GCN5 on apical dominance. Indeed, the RGA represses GA-induced apical dominance [43].

GCN5 is essential in flower development, especially in reproductive organs, stamens, and the gynoecium [7,10,20]. GCN5 promotes stamen filament elongation in the early formed flowers ([10], this study) by affecting the expression and the histone acetylation levels on late-stage biosynthetic genes, GA20ox2 and GA3ox1, as well as the GA-inactivating genes. GA3ox1 is expressed in the early flowers’ stamen filament [38]. Our data suggest that the positive role of GCN5 on stamen filament growth is suppressed by RGA action. This scenario does not arise from changes in the gene expression and histone acetylation levels of GA biosynthesis. However, the effect of the RGA is concentrated on the expression level of GA catabolism genes, GA receptor GID1b, and the GAI DELLA protein, suggesting that the GCN5 effect on those genes is mediated by RGA action.

Moreover, the effect of GCN5 on histone H3K14 acetylation levels in the GAI promoter is restored by the RGA, either by recruiting another histone acetyltransferase or by inhibiting the action of a histone deacetylase. During flower initiation, the H3K14 acetylation levels in the GAI locus are positively affected by ADA3a, a component of the SAGA complex [17]. HDA15 is recruited in the promoter GA biosynthesis genes and represses their expression [44]. Beyond the gibberellin effect on stamen filament elongation, another plant hormone, jasmonic acid, is also involved [23]. The RGA and RGL2 are critical for the inhibition of stamen development [45] by interacting with transcription factors MYB21 and MYB24, acting as a central synergistic node of GA and JA signaling on stamen filament interaction [46]. The opposite activities of GCN5 and HDA6 regulate TPL acetylation and repressor activity to determine the transcription of JA-responsive genes [47]. This scenario could also explain the effect of the RGA on GCN5 action on stamen elongation.

Our data also suggest that the RGA does not repress several GCN5 actions. Those include the promotion of root elongation in seedlings, the effect of GCN5 leaf serration patterning and development, and the positive role of fruit elongation and development. Interestingly, the RGA and RGL2 could not suppress the ADA2b action, suggesting that ADA2b acts downstream of DELLA action. Furthermore, GCN5 and ADA2b could have distinct mechanistic regulations in GA signaling. Although it is unclear if there is a physical interaction between the RGA and GCN5 or other members of the SAGA complex, recently, the RGA was found to interact with H2A to form a complex between transcription factors, the RGA and H2A complex [48]. In the future, it is necessary to provide mechanistic evidence of the possible DELLA–histone acetylation interaction and how this regulates developmental stages, tissues, and cell type differentiation.

## 4. Materials and Methods

### 4.1. Plant Material and Plant Growth Conditions

In this study, Arabidopsis thaliana ecotypes Col-0 and Ws-2, as well as single mutants *ada2a-3*, *ada2b-1*, *gcn5-1*, *gcn5-6/hag1-6*, and *prz1-1*, described in [9,18,19,49], were used. Seeds were first disinfected with 30% bleach for 5 min and then stratified at 4 °C for three days. The seeds were sown in Petri dishes containing Gambrog B5 nutrient medium (Douchefa, Haarlem, The Netherlands) with pH 5.7, 1% sucrose, and 0.8% *w*/*v* phyto-agar (Douchefa, Haarlem, The Netherlands), and then transferred to a growth chamber with a constant temperature of 20 °C and a constant light intensity (100 μmol*m^−2^*s^−1^). The plant growth photoperiod was 16 h of light and 8 h of darkness.

### 4.2. Hypocotyl and Root Elongation Measurements

After five days of germination, seedlings were transplanted into Petri dishes containing Gamborg B5 medium (Duchefa, Haarlem, The Netherlands) and different concentrations of gibberellic acid (0 μM GA_3_, 2.5 μM GA_3_ and 10 μM GA_3_) without sucrose. These Petri dishes were placed perpendicular to the light. Photos were taken for four consecutive days and processed with ImageJ https://imagej.net/ij/ (NIH, Bethesda, MD, USA). The experiments were repeated thrice, and 20 seedlings per genotype per treatment were used.

### 4.3. Genetic Analysis

The *Arabidopsis thaliana* (L) Heynh. *gcn5-6 (hag1-6)* and *ada2b-1* mutants were previously described [49,50]. The *rga-t2* was obtained by the European Arabidopsis Stock Centre (NASC) and described [51]. The *gcn5-6;rga-t2* and the *ada2b-1;rga-t2* double mutants were created using pollen from *rga-t2* homozygous mutants to fertilize *gcn5-6* and *ada2b-1* gynoecium. The resulting F1 generation was self-fertilized, and the segregating F2 population was genotyped using PCR-based methods. The enzyme ExTaq DNA polymerase (Takara, Shiga, Japan) was used to confirm the double mutants; the primers are listed in Supplemental Table S4. The double mutants were backcrossed to Ws-2 or Col-0 background for at least four generations. Kanamycin resistance of *ada2b-1* alleles was used when applicable to facilitate selection.

### 4.4. Gene Expression Assays

For the RT-qPCR expression analysis, whole inflorescences from Col-0, *rga-t2*, *gcn5-6*, and *rga-t2;gcn5-6* plants were collected when the first one or two open flowers emerged and flash-frozen in liquid nitrogen. The frozen tissue was preserved at −70 °C. Five different harvests were made, and three were used for RNA extraction using the Nucleospin^®^ RNA Plant kit (Macherey-Nagel, Duren, Germany). RNA quality and quantity were assessed using 1.5% gel electrophoresis and NanoDrop 2000 (Thermo Fischer Scientific, Waltham, MA, USA). The PrimeScriptTM 1st strand cDNA synthesis kit (Takara, Shiga, Japan) was used for reverse transcription. In three independent biological repeats, reverse transcription was performed using 0.5 μg of total RNA. Quantitative reverse-transcription polymerase chain reactions (RT-qPCRs) were prepared with the AMPLIFYME SG Universal Mix (AM02) (BLIRT SA, Gdansk, Poland) or the Luna^®^ Universal qPCR Master Mix (New England Biolabs, Ipswich, MA, USA) using the ABI StepOnePlus™ system (Applied Biosystems, Foster City, CA, USA). Three technical repeats were run for each sample. The At4g26410 or the PDF2 genes were used as endogenous controls (Supplemental Table S4). Data were analyzed with the ΔΔCt method using StepOne Software 2.1. Statistical analysis was performed using R (Integrated Development Environment (IDE) RStudio version 2023.03.0+386, known as “Cherry Blossom” [52].

### 4.5. Chromatin Immunoprecipitation Assays

Whole inflorescences of Col-0, *rga-t2*, *gcn5-6*, and *rga-t2;gcn5-6* plants were collected when the first one or two open flowers emerged. The tissue was fixed in 1% formaldehyde under vacuum for 15 min, and the crosslinking reaction was terminated with 0.125M glycine for 5 min under vacuum. Samples were stored at −70 °C. Approximately 300 mg of tissue from each genotype was ground in liquid nitrogen to a fine powder, and nuclei were extracted and lysed in the presence of 1% SDS. Chromatin was sheared into 200 to 1000 bp fragments using Fisherbrand™ Model 505 Sonic Dismembrator (Fischer Scientific, Waltham, MA, USA) with the following parameters: sonication for 10 s and stopping for 50 s at 50% power five times. Chromatin was diluted ten times before the immunoprecipitation, with antibodies against acetylated histone H3K14 (Anti-Histone H3 (Lys14), EMD Millipore #07-353, Burlington, MA, USA) and non-acetylated histone H3 #ab1791 (Abcam, Cambridge, UK). The precipitation was performed using agarose-protein A beads (Cell Signaling, Danvers, MA, USA). The elution of chromatin attached to the beads was made at 65 °C with 1% SDS and 0.1M NaHCO3. Formaldehyde crosslinking was reversed in the presence of 200 mM NaCl at 65 °C overnight, followed by proteinase K (Sigma-Aldrich, St Louis, MI, USA) treatment. The DNA was isolated using a commercially available PCR clean-up kit (Macherey-Nagel, Duren, Germany). Immunoprecipitated DNA was diluted in water and analyzed with qPCR using specific primers (Supplemental Table S4). Luna^®^ Universal qPCR Master Mix (New England Biolabs, Ipswich, MA, USA) and the ABI StepOnePlus™ system (Applied Biosystems, Foster City, CA, USA) were utilised for the qPCR assays. Ten-fold serial dilutions of input Col-0 were used to create a standard curve. All data obtained with q-PCR were presented as a percentage of input. The value of each immunoprecipitated sample was normalized to the input. The ratio of acetylated H3K14 to H3 values of each genotype is presented in the graphs. The immunoprecipitation assays were performed in three independent biological repeats. Statistical significance was calculated using Student’s *t*-test in R [52].

### 4.6. List of Genes Used in This Work

Table 1 presents the official gene names used in the manuscript.

## 5. Conclusions

**Figure 8:**
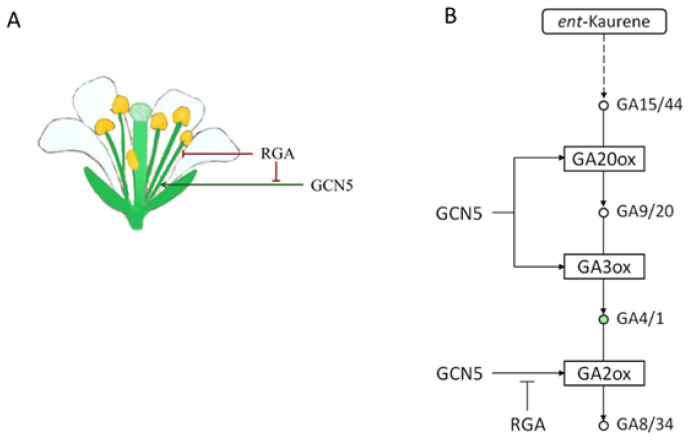
The genetic interaction between RGA and GCN5 on stamen filament growth (**A**). GCN5 is a positive regulator of GA biosynthesis and catabolism. RGA suppresses GCN5 action on GA2 oxidases (**B**).

In conclusion, our findings add essential evidence to our current understanding of mechanisms involving the interaction of gibberellins and histone acetylation. We have shown that the GA signaling repressor RGA suppresses the histone acetyltransferase GCN5 action on stamen filament elongation by affecting histone acetylation-mediated gene expression on GA catabolism and signaling (Figure 8A,B). Additional molecular and genetic studies may further dissect the role of GCN5 in the development of reproductive organs, and their complex interaction and biochemical analyses may reveal the mechanisms by which histone acetylation affects GA signaling.

## Figures and Tables

**Table 1:** The complete genes name, the gene symbol, and the locus name of the gene used in this manuscript.

Gene Symbol	Gene Full Name	Locus
*ADA2a*	*ALTERATION-DEFICIENCY IN ACTIVATION 2a*	AT3G07740
*ADA2b*	*ALTERATION-DEFICIENCY IN ACTIVATION 2b*	AT4G16420
*GCN5*	*GENERAL CONTROL NON-DEREPRESSIBLE 5*	AT3G54610
*RGA*	*REPRESSOR OF GA1-3*	AT2G01570
*RGL1*	*RGA-LIKE 1*	AT1G66350
*RGL2*	*RGA-LIKE 2*	AT3G03450
*GAI*	*GA INSENSITIVE*	AT1G14920
*GA3ox1*	*GIBBERELLIN 3-OXIDASE 1*	AT1G15550
*GA20ox2*	*GIBBERELLIN 20 OXIDASE 2*	AT5G51810
*GA2ox4*	*GIBBERELLIN 2 OXIDASE 4*	AT1G47990
*GA2ox6*	*GIBBERELLIN 2 OXIDASE 6*	AT1G02400
*GA2ox7*	*GIBBERELLIN 2 OXIDASE 7*	AT1G50960
*GA2ox8*	*GIBBERELLIN 2 OXIDASE 8*	AT4G21200
*GID1b*	*GA INSENSITIVE DWARF1b*	AT3G63010

## Data Availability

The data that support the findings of this study are available from the corresponding author upon reasonable request.

## Supplementary Materials

The following supporting information can be downloaded at: https://www.mdpi.com/article/10.3390/ijms25126757/s1.
