# Supplementary material for: Gibberellin Signaling through RGA Suppresses GCN5 Effects on Arabidopsis Developmental Stages
Source: Int J Mol Sci. 2024 Jun 19;25(12):6757. doi: 10.3390/ijms25126757 (PMC11203840; doi:10.3390/ijms25126757)
Supplement: Supplementary file 1 [file ijms-25-06757-s001.zip › ijms-3047008-supplementary.pdf]

Supplementary Materials (Balouri et al.)

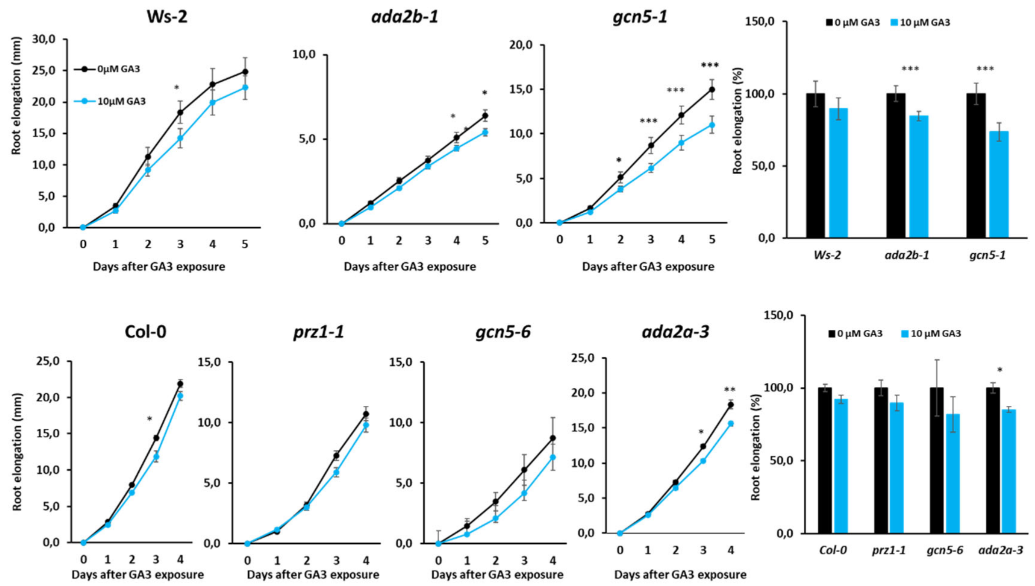

Figure S1. The effect of GA_3 on root elongation of Ws-2, *ada2b-1*, *gcn5-1*, Col-0, *prz1-1*, *gcn5-6*, and *ada2a-3* seedlings. Sensitivity of Ws-2, *gcn5-1* and *ada2b-1*, and Col-0, *gcn5-6*, *prz1-1* and *ada2a-3* mutant seedlings after three days of exposure to 10μM GA_3. Asterisks *, ** and *** indicate statistically significant differences from 0μM GA_3 using Student's t-test for $P < 0.05$, $P < 0.01$ and $P < 0.001$, respectively (n=90 plants).

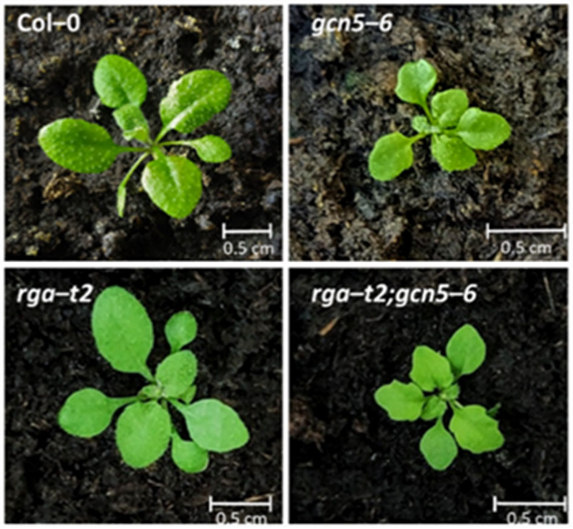

Figure S2. The effect of GCN5 and RGA on leaf development. Rosette development of twenty days old Col-0, *gcn5-6*, *rga-t2* and rga-*t2;gcn5-6* plants.

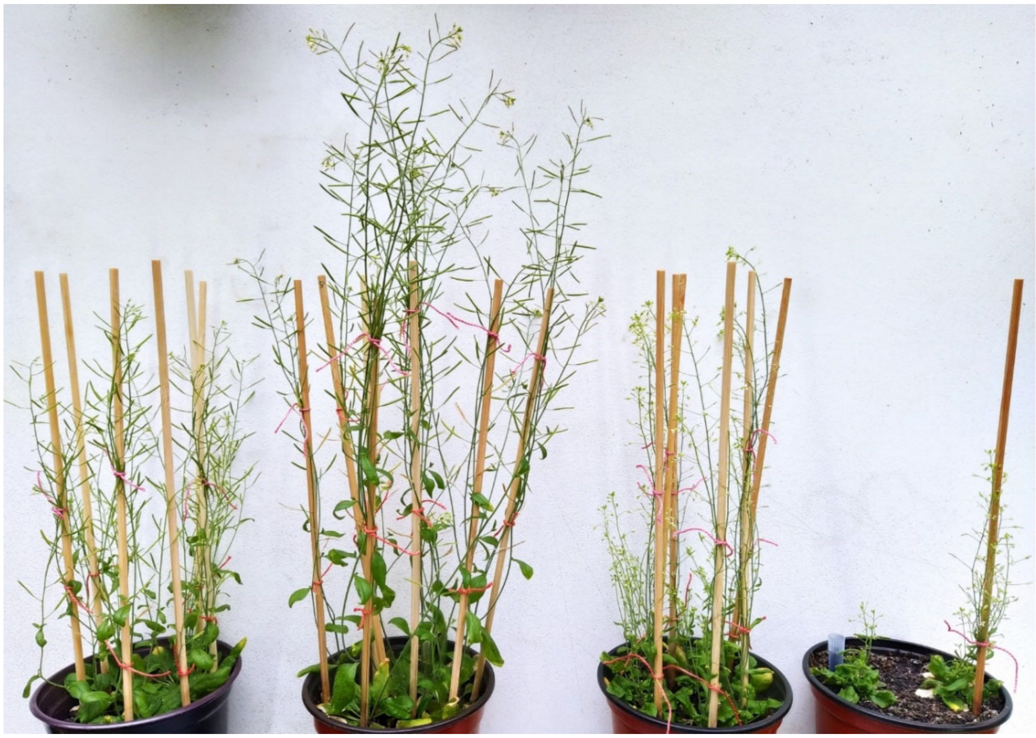

Figure S3. Col-0, *rga-t2*, *gcn5-6* and *rga-t2;gcn5-6* mutant plants after 57 days of growth.

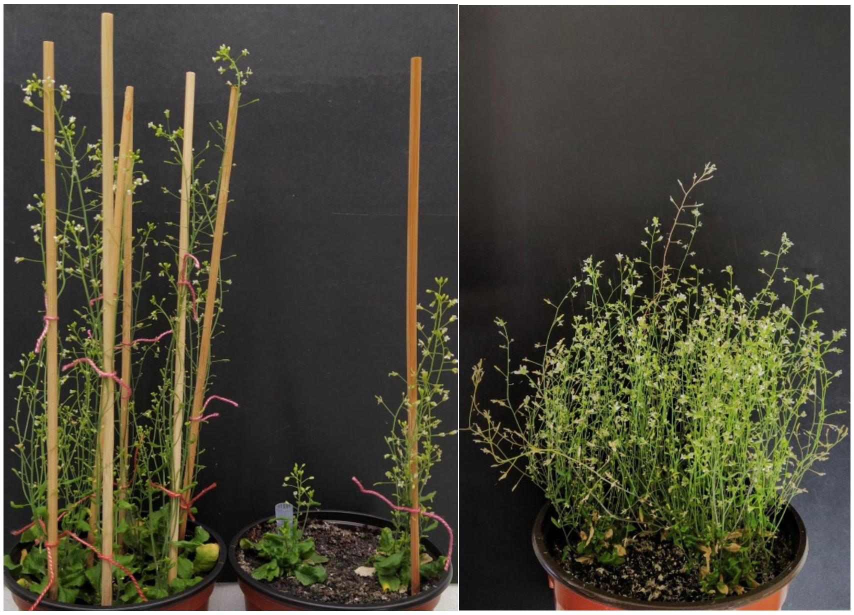

Figure S4. A) *gcn5-6* and *rga-t2gcn5-6* mutant plants after 57 days of growth and B) *gcn5-6* mutant plants after 90 days of growth.

Table S1. The effect of GCN5 and RGA on the number of lateral inflorescences.

Genotype	Col–0	*rga–t2*	*gcn5–6*	*rga–t2;gcn5–6*
**Mean ± standard deviation**	1,9 ± 0,38	2,5 ± 0,53	1,7 ± 0,48	2,6 ± 0,51
**N**	7	8	10	16
**P from Col–0**	-	0,028	0,37	0,004
**P from double**	-	0,66	0,0002	-

Table S2. The effect of GCN5 and RGA on the number of secondary inflorescences.

Mutant	*gcn5–6*	*rga–t2;gcn5–6*
**Mean ± Standard Deviation**	15,6 ± 5,28	4,1 ± 1,20
**N**	11	16
P	0,0001	

Table S3. The effect of GCN5 and RGA on the number of stamens in early flowers.

Genotype	*Col–0*	*rga–t2*	*gcn5–6*	*rga–t2;gcn5–6*
**Mean ± Standard Deviation**	5,6 ± 0,52	5,4 ± 0,51	6,1 ± 0,35	7 ± 0,85
**N**	8	16	8	12
P from Col–0	-	0,4085	0,0407	0,0007
P from double	-	0,0001	0,0138	-

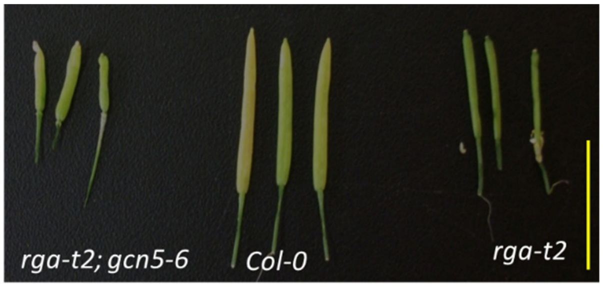

Figure S5. *rga* suppress *gcn5* infertility. Siligues of Col-0, *rga-t2* and *rga-t2;gcn5-6*, The bar equals 1,5 cm.

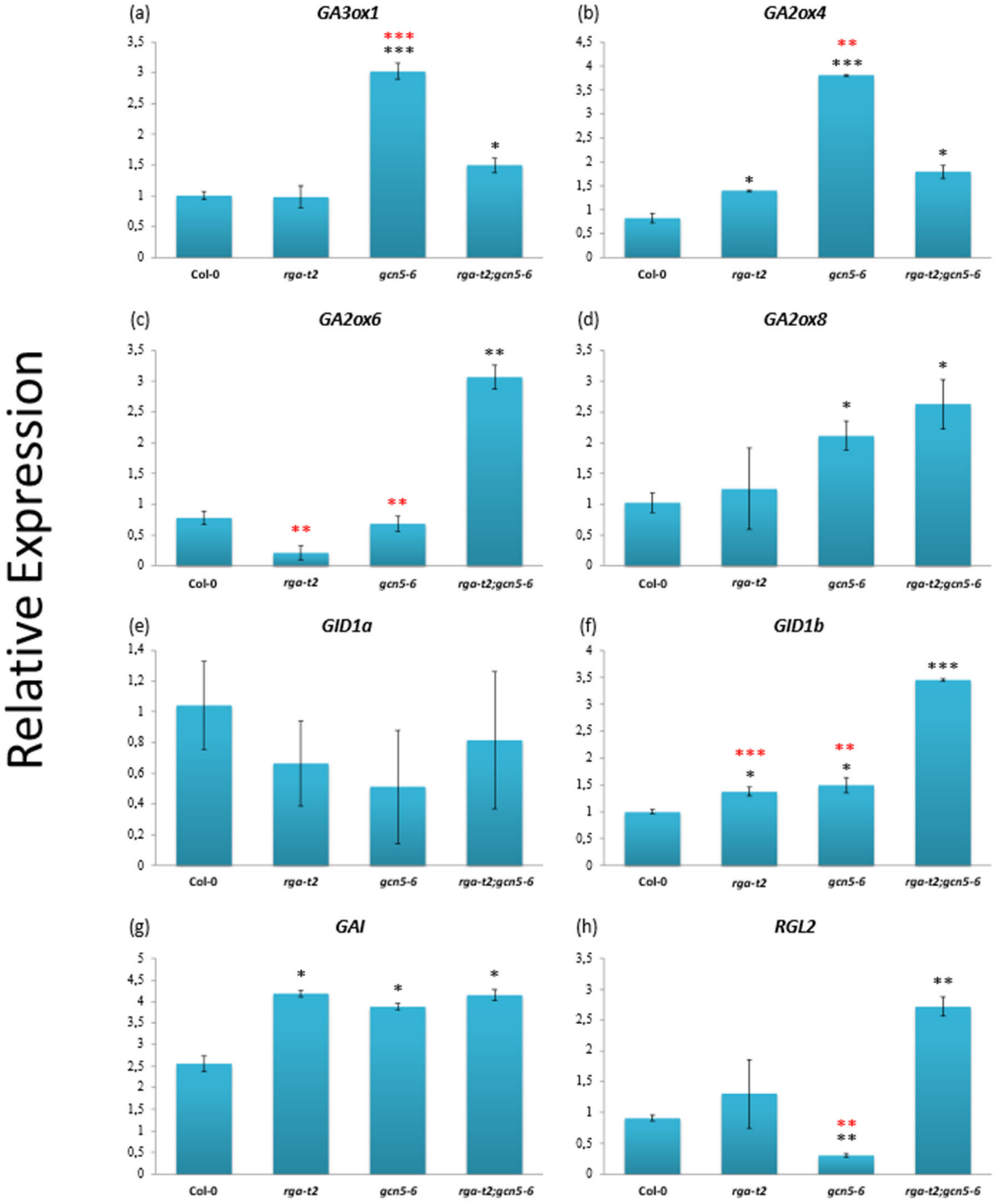

Figure S6. Expression of gibberellin biosynthesis gene *GA3ox1* (a), gibberellin catabolism genes *GA2ox4*, *GA2ox6*, and *GA2ox8 (b*,*c* and *d)* GA-receptors *GID1a* and *GID1b (e* and *f)*, and DELLA genes *GAI* and *RGL2 (g* and *h)* in hypocotyl of *Arabidopsis thaliana.* Error bars show Standard Error. Asterisks show statistical significance compared to Col–0 and *rga–t2;gcn5–6* double mutant (red astericks), using Student's *t*–test: *, $P < 0.05$, **, $P < 0.01$, and ***, $P < 0.001$.

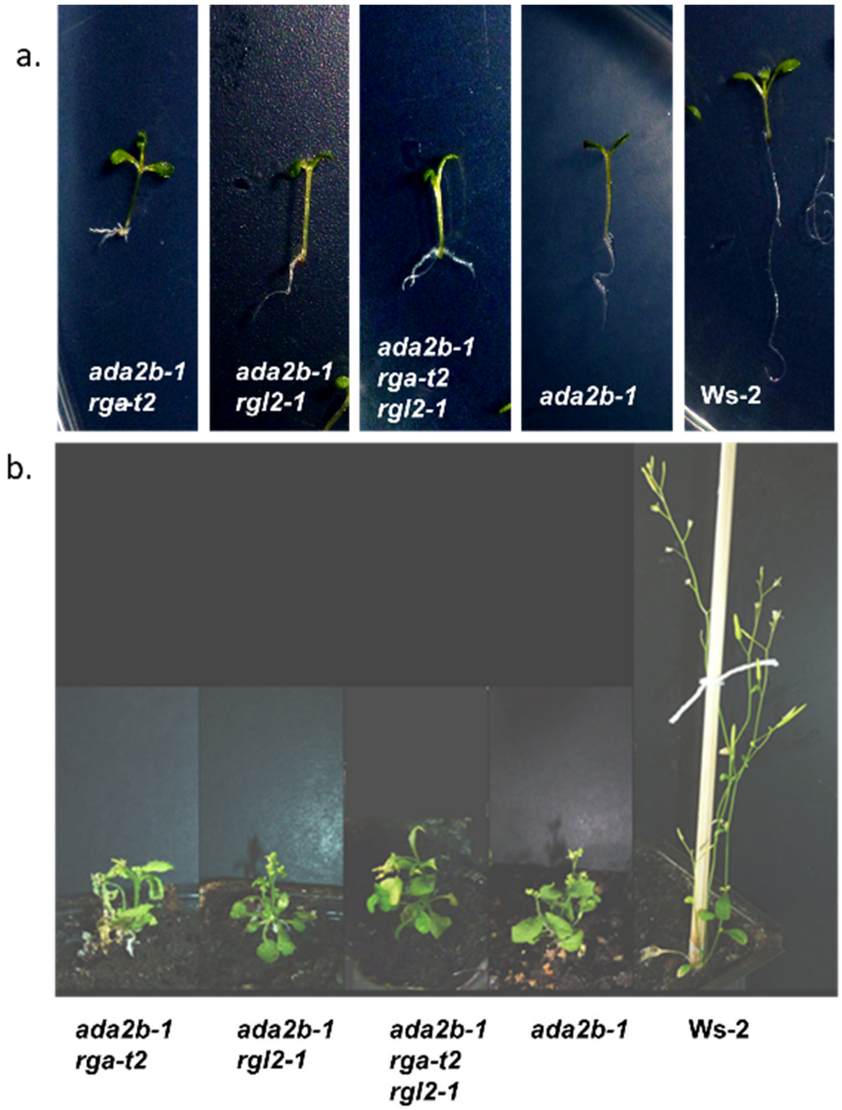

Figure S7. Characterization of *ada2b;rga, ada2b;rgl2,* double mutants and *ada2b;rga;rgl2* triple mutants at seedlings stage *(a)* and after 42 days of growth in long days conditions *(b).*

Table S4. Primers used in this study.

Target	Primer name	Sequence	Assay
At4g26410	KVDW034 (F)	5′-GAGCTGAAGTGGCTTCCATGAC-3′	RT-qPCR
	KVDW035 (R)	5′-GGTCCGACATACCCATGATCC-3′	
GA2ox4	KVDW101 (F)	5′-CACCGTCCGATCAATTCTTT-3′	RT-qPCR
	KVDW102 (R)	5′-ATCTGAGGGTCGGTGTGTTC-3′	
	KVDW103 (F)	5′-AGTGGCGTTGGTCAAATAGG-3′	

GA2ox6	KVDW104 (R)	5′-AGGCAGTCACCGACCAATAC-3′	RT-qPCR
GA2ox7	KVANA132 (F)	5′-TCGATTTTCGGAAGCTCATT-3′	ChIP
	KVANA133 (R)	5′-GCTTTATGCCTGAGGTTTGG-3′	
	KVDW097 (F)	5′-TTCTGATGATCGCAACAACC-3′	RT-qPCR
	KVDW098 (R)	5′-CGTTCACTTGTTTCCCCAGT-3′	
GA2ox8	KVANA136 (F)	5′-TGTGTCTCTCCTCACAACACG-3′	ChIP
	KVANA137 (R)	5′-GAATGGTGGATCCATGAGAAG-3′	
	KVDW099 (F)	5′-GAGGAGGTGGAGCTTCCTGT-3′	RT-qPCR
	KVDW100 (R)	5′-TCTCGCAATCGCTTCCTTAC-3′	
GA3ox1	KVANA124 (F)	5′-CATAGTTTGACAACCCATCATGT-3′	ChIP
	KVANA125 (R)	5′-TATAGAGGTCCCGCCATTTC-3′	
	KVDW057 (F)	5′-AGGAGAAGGAGCAGCGGAGAAGAGGAG -3′	RT-qPCR
	KVDW058 (R)	5′-CATCCCATTCACCTCCCACACTCTCACATAC-3′	
GA20ox2	KVANA128 (F)	5′-GAAATCCCATGTGGCAAAAA-3′	ChIP
	KVANA129 (R)	5′-AGCAAGCTTCAAGTCATGTTAGTG-3′	
	KB312 (F)	5′- TCCAACGATAATAGTGGCT -3′	RT-qPCR
	KB313 (R)	5′- TTGGCATGGAGGATAATGA -3′	
GAI	KVANA93 (F)	5′-GGTGTGTGTGTGATTTTCAGC-3′	ChIP
	KVANA94 (R)	5′-CCGATGAATCATGGATCAGA-3′	
	KVDW055 (F)	5′-AACTCGGCATGTTGTCCTG-3′	RT-qPCR
	KVDW056 (R)	5′-AAAGCGCGTGAACGAGAC-3′	
GCN5	KB118 (R)	5′-TTGAGATTTAGCACCAGATTGGAGACCTG-3′	PCR genotyping
	KB290 (F)	5′-CTCCAACGATGAACTCGAGAG-3′	
gcn5-6	KB290 (F)	5′-CTCCAACGATGAACTCGAGAG-3′	PCR genotyping
	KV181/ LBa1 (R)	5′-TGGTTCACGTAGTGGGCCATC G-3′	
GID1a	KVCB-GID1AF (F)	5′-TTAAGAGCGGGTGAATCGGG-3′	RT- qPCR
	KVCB-GID1AR (R)	5′-CCGTTCTCTCATTCCCACCA-3′	
GID1b	KVCB-GID1BF (F)	5′-CTACCAACCTGCGTCTCTCC-3′	RT-qPCR
	KVCB-GID1BR (R)	5′-TGAGTGAAGCTGCCTCCATG-3′	
PDF2	KVI126 (F)	5′-AGGCAGAAGTTCGGATAGCA-3′	ChIP
	KVI127 (R)	5′-CAGGGAAGAATGTGCTGGAT-3′	
	KVI192 (F)	5′-TAACGTGGCCAAAATGATGC-3′	RT-qPCR
	KVI193 (R)	5′-GTTCTCCACAACCGCTTGGT-3′	

RGA	KB395 (F)	5′-GCCGGAGCTATGAGAAAAGTG-3′	PCR genotyping
	KB396 (R)	5′-AAGAATTTTAAACAAGTGAACG-3′	
rga–t2	KB395 (F)	5′-GCCGGAGCTATGAGAAAAGTG-3′	PCR genotyping
	KB404 (R)	5′-CCGGTATATCCCGTTTTCG-3′	
RGL2	KVDW053 (F)	5′-CTGCGTTTCCAAAGGAAGAG-3′	RT-qPCR
	KVDW054 (R)	5′-GTCGGATCCTCTTGCTGCTA-3′	
	KB399 (F)	5′-GCT GGT GAA ACG CGT GGG AAC A-3′	PCR genotyping
	KB400 (R)	5′-ACG CCG AGG TTG TGA TGA GTG-3′	
